# Beraprost sodium protects against chronic brain injury in aluminum-overload rats

**DOI:** 10.1186/s12993-014-0051-7

**Published:** 2015-02-07

**Authors:** Yongquan Pan, Lijuan Yu, Wenjuan Lei, Yuanxin Guo, Jianfeng Wang, Huarong Yu, Yong Tang, Junqing Yang

**Affiliations:** Department of Pharmacology, Chongqing Medical University, the Key Laboratory of Biochemistry and Molecular Pharmacology, Chongqing, 400016 China; Laboratory Animal Center, Chongqing Medical University, Chongqing, 400016 China; Department of Basic College, Chongqing Medical University, Chongqing, 400016 China; Department of Pharmacology, Chongqing Medical University, Chongqing, 400010 China

**Keywords:** Chronic brain injury, Beraprost sodium, PGI2 level, PGIS, IP receptor

## Abstract

**Background:**

Aluminum overload can cause severe brain injury and neurodegeneration. Previous studies suggest that prostacyclin synthase (PGIS) expression and prostacyclin receptor (IP) activation are beneficial for treatment of acute traumatic and ischemic brain injury. However, the potential value of PGIS/IP signaling pathway to chronic brain injury is still unclear. In this study, we investigated the change of PGIS/IP signaling pathway and the effect of beraprost sodium (BPS) on chronic brain injury in chronic aluminum-overload rats.

**Methods:**

Rat model of chronic cerebral injury was established by chronic intragastric administration of aluminum gluconate(Al^3+^ 200 mg/kg per day,5d a week for 20 weeks). The methods of ELISA, qRT-PCR and Western blotting were used to detect the PGI2 level and the PGIS and IP mRNA and protein levels in hippocampi of chronic aluminum-overload rats, respectively. Rat hippocampal superoxide dismutase (SOD) activity and malondialdehyde (MDA) content also were measured. The effects of BPS (6, 12 and 24 μg⋅kg^-1^) on brain injury in chronic aluminum-overload rats were evaluated.

**Results:**

Compared with the control group, PGIS mRNA expression, PGI2 level, and the IP mRNA and protein expressions significantly increased in hippocampi of chronic aluminum-overload rats. Administration of BPS significantly improved spatial learning and memory function impairment and hippocampal neuron injury induced by chronic aluminum overload in rats. Meanwhile, administration of BPS resulted in a decrease of PGI2 level and downregulation of PGIS and IP expressions in a dose-dependent manner. Aluminum overload also caused a decrease of SOD activity and an increase of MDA content. Administration of BPS significantly blunted the decrease of SOD activity and the increase of MDA content induced by aluminum overload in rats.

**Conclusions:**

BPS has a significant neuroprotective effect on chronic brain injury induced by aluminum overload in rats. Remodeling the balance of PGIS/IP signaling pathway and inhibition of oxidative stress involve in the neuroprotective mechanism of BPS in aluminum-overload rats. The PGIS/IP signaling pathway is a potential therapeutic strategy for chronic brain injury patients.

## Background

Aluminum, which accounts for about 8% of the earth crust mass, is the most widely distributed metal in air, water and soil and is extensively used in modern daily life. Therefore, we unavoidably take in aluminum by various routes. However, the physiological action of aluminum to humans is not clear. It is well known that aluminum overload can cause severe brain injury and neurodegeneration since the aluminum toxicity to humans was first reported at early 1970s. In particular, aluminum was detected in senile plaques and neurofibrillary tangles in brain tissue of Alzheimer’s disease (AD) patients, indicating that aluminum neurotoxicity may be involved in the pathogenesis of AD [1,2]. Despite some debated studies, the majority of scholars think there is a close relationship between aluminum exposure and the risk of developing AD [3]. Aluminum could play a role in chronic brain damage and neurodegeneration by enhancing oxidative stress and inflammation [4]. However, the mechanism of aluminum neurotoxicity is not well understood and should be further clarified.

Proinflammatory cytokines including tumor necrosis factor (TNF)-α, interleukins (ILs) and transforming growth factor (TGF)-β make large contributions to the neuroinflammatory response in patients with chronic brain injury. IL-1 and TNF-α can activate many cerebral signaling pathways including the arachidonic acid (AA) metabolic cascade [5]. Under the action of cyclooxygenase (COX), AA can be converted to bioactive prostaglandin (PG)H2. PGH2 serves as the substrate for multiple specific prostaglandin synthases (PGS) and can be metabolized to PGD2, PGE2, PGF2a, PGI2 or thromboxane (TX)A2. COX has at least two isoforms, the constitutive COX-1 and the inducible COX-2. COX-1 is prominently expressed in microglia of human brain [6]. COX-2 is highly expressed in the neuronal cell bodies and dendritic regions of hippocampus, cerebral cortex, and amygdale. COX-2 can be rapidly induced following traumatic brain injury, acute cerebral ischemia, seizures, and neurodegeneration [7]. Our previous studies also demonstrate that aluminum significantly induced brain injury and COX-2 overexpression, and the COX-2 inhibitor protected against chronic brain injury in rats [8]. However, celecoxib and naproxen could neither prevent AD nor improve cognitive function of AD patients in some randomized trials [9,10]. A further clinical trial shows that therapeutic effects of NSAIDs differ at different stages and COX inhibitors have serious adverse effects at later stages of AD [11]. Despite the failed clinical treatment trial and the terminated prevention trial for NSAIDs, the specific COX downstream signaling pathway has become a focus in neurological research to observe its potential therapeutic values for modification of chronic brain injury and for avoidance of the toxicities of NSAIDs.

Prostacyclin, also known as PGI2, is derived from PGH2 under the action of prostacyclin synthase (PGIS). Through acting mainly on the membrane-bound prostacyclin receptor (IP), PGI2 and its analogues play lots of physiological and pharmacological functions. Combined gene transfer of COX-1 and PGIS could increase PGI2 level and decrease cerebral infarct volume in rats with ischemic reperfusion [12]. Administration of PGI2 significantly reduced the cortical lesion of rats with traumatic brain injury [13]. IP knockout mice developed more severe cortical lesion than wild type mice [14].

These studies suggest that activation of IP is beneficial for acute cerebral ischemic insult and traumatic brain injury. However, the PGI2 level, and the mRNA and protein expressions of PGIS and IP in brains of rats with chronic brain injury have not been characterized. Moreover, there is no report about the effect of PGIS/IP signaling pathway activation on cerebral damage in rat with chronic brain injury.

We previously established a rat model of chronic brain injury *via* chronic intragastric administration of aluminum gluconate. Administration of aluminum gluconate caused an increase of hippocampal aluminum level (21.53 ± 2.90 *vs* 61.17 ± 9.25 μg/g) and learning and memory function disorder [15,16]. The present study was designed to investigate the effects of beraprost sodium (BPS, an agonist of IP) on chronic brain injury and to primary explore neuroprotective mechanism of BPS through detecting the change of hippocampal neuronal PGI2 content, and the expressions of PGIS and IP at mRNA and protein levels in chronic aluminum-overload rats.

## Methods

### Reagents

RNAiso Plus total RNA extraction kit, and PrimeScipt RT regeant Kit with gDNA Eraesr (Perfect Real Time) (TAKARA Bio(Dalian) CO.,LTD, Dalian); iQ SYBR Green Real-Time PCR Supermix (BioRad, USA); mouse antirat β-actin monoclonal antibodies, and goat antimose horseradish peroxidase secondary antibodies (Zhongshan Goldbridge Technology Co., Ltd., Beijing); rabbit antirat IP monoclonal antibodies, and goat antirabbit horseradish peroxidase secondary antibodies (Cayman, USA); BCA protein detection kit, and BeyoECL chemiluminescence detection kit (Beyotime Institute Biotechnology, Shanghai) were used. BPS (Beijing Tide Pharmaceutical CO.,LTD, Beijing) was prepared with 0.5% sodium carboxy methyl cellulose (CMC-Na) before use.

### Animals and experimental protocol

A total of 75 male Sprague Dawley rats, weighing 200-250 g and aged 8 weeks, were supplied from the Laboratory Animal Center of Chongqing Medical University (No. SCXK (Yu) 2007-0001). They were housed in standard conditions of 50 ± 2% humidity, 22 ± 2°C, and 12 h light/dark cycles (light from 8:00-20:00). All experimental procedures on animals were approved by Institutional Animal Ethics Committee of Chongqing Medical University. The research was also conducted in accordance with the guide for the Care and Use of Laboratory Animals promulgated by the United States National Institutes of Health. The 75 rats were randomly divided into five group depending on body weight (each *n* = 15): a control group, an aluminum-treated group (Al-overload group), and three BPS-treated groups (6, 12, and 24 μg⋅kg^-1^ BPS, marked as BPS-6 group, BPS-12 group and BPS-24 group, respectively).

Rats in the Al-overload group received chronic intragastric administration of aluminum gluconate (Al^3+^ 200 mg/kg per day), followed by administration of 0.5% CMC-Na 2 h later. Aluminum was administrated 5 days per week for 20 continuous weeks. Two hours later after aluminum administration, rats in BPS-treated groups were intragastrically administered with BPS (6, 12, and 24 μg⋅kg^-1^). The control group was treated with an equal volume of sodium gluconate followed by an equal volume of 0.5% CMC-Na 2 h later [8,16].

### Morris water maze test

On the next day after aluminum administration was stopped, the rats in each group were subjected to spatial learning and memory test using a DMS-2 Morris water maze (Institute of Materia Medica, Chinese Academy of Medical Sciences, Beijing, China) with a diameter of 1.5 m, height of 0.5 m, water depth of 0.4 m and temperature of 23 ± 2°C. The rats were subjected to study how to navigate the maze for four days. On the fifth day, spatial memory was tested as previously reported study [17]. The rats in each group received a probe trial in which the platform was removed. The time for the rat to pass through the place, where the platform was previously placed, was called as the time for exploring platforms (exploring time).

### Pathomorphological observation

Six days after aluminum administration was completed, four rats from each group were selected for histopathological observation. According to a reported method [17], the rats were intraperitoneally anesthetized with 4% chloral hydrate (1 mL/100 g) and transcardially perfused with 150 mL of 0.9% saline containing 375U heparin, followed by 200 mL of 4.0% paraformaldehyde fixing solution containing 0.1 M phosphate buffer solution (PBS, pH 7.2). The cerebral tissue was isolated and made into coronal sections of 4 μm thicknesses for hematoxylin and eosin staining (H.E). Neuronal pathological change in rat hippocampus was observed under light microscopy. Ten consecutive fields from the dorsal hippocampal CA1 subfield of each section were selected, and then intact neurons were counted by a microscope at 400× magnification and the extent of cell death was estimated. Dead neuron is characterized by eosinophilic change including nerve cell became a deep and red cell with nuclear pyknosis and nucleoli disappear under light microscope.

### Measurement of malondialdehyde (MDA) content and superoxide dismutase (SOD) activity

Four rat brains from each group were removed six days after aluminum administration was completed. Hippocampus was homogenized with saline by a weight-volume ratio of 1:9. The homogenate was centrifuged and the supernatant was collected for detection of SOD activity and MDA content according to the manufacturer’s manual (Jiancheng Bioengineering Ltd, Nanjing, China). Protein content was measured by biuret spectrophotometry method.

### Detection of PGI2 level

Hippocampal homogenate from each group (n = 4) was prepared with saline by a weight-volume ratio 1 : 9, followed by centrifugation at 8000 g × 10 min under 4°C. The supernatant was collected for detection of PGI2 level according to the manual of the enzyme-linked immuno- sorbent assay (ELISA) kit (Cayman, USA). Since PGI2 can be easily metabolized, the level of 6-keto-prostaglandin F1α (6-keto-PGF1α), a metabolite of PGI2, was detected. In order to avoid the post-mortem production of prostanoids from oxylipids, the indomethacin, a nonselective inhibitor of COX-1 and COX-2, was added according to the manual of the ELISA kit.

### Quantitative real-time polymerase chain reaction (qRT-PCR) analysis of PGI2 and IP mRNA expressions

Rat brain hippocampi from each group (n = 4) were removed for qRT-PCR analysis of PGI2 and IP following the learning and memory function test.

RNA was isolated and prepared by using an RNAiso Plus total RNA isolation kit (TAKARA). PrimeScipt RT regeant Kit with gDNA Eraesr (TAKARA) was used for cDNA synthesis. The total reaction volume for erase of genome DNA was 10 μL, including 2 μl of 5 × DNA Eraser Buffer, 1 μl of gDNA Eraser, and 1 μg of total RNA with RNase-Free ddH_2_O. Condition for erase of genome DNA was 42°C for 2 min, followed by 4°C for 10 min. The total reaction volume for RT was 20 μL, including 4 μl of 5 × PrimeScript Buffer, 1 μl of PrimeScript RT Enzyme Mix I, 1 μg of RT Primer Mix, and 10 μl of reaction liquid with RNase Free ddH_2_O. The RT conditions were 37°C for 15 min, 85°C for 4 sec and 4°C for 10 min. The synthesized cDNA was the template for the qRT-PCR performed on the CFX RT-PCR detection system (Bio-Rad). The following primer sequences were used: forward 5’-GCTACCTGACCCTGTATGGAGT-3’, reverse 5’-GTCTTTATCCCCCACTGACAAG-3’ for PGIS; forward 5’-CTGCTGGTGACCTCCATCTTCT-3’, reverse 5’-CTGCGTGAATCCTCTGATCGT-3’ for IP; forward 5’-ACAGCAACAGGGTGGTGGAC-3’, reverse 5’-TTTGAGGGTGCACGAACTT-3’ for glyceraldehyde phosphate dehydrogenase (GAPDH). The total reaction volume for qRT-PCR was 20 μL, including 10 μl of iQ SYBR Green RT-PCR Supermix (BioRad), 0.6 μl of forward/reverse primers (10 μM), 2 μl of cDNA, and 7.4 μl of RNase-Free ddH_2_O. The qRT-PCR conditions were 95°C for 3 min, 95°C for 10 sec and annealing temperature 57.9°C for PGIS (62.3°C for IP and 63.5°C for GAPDH) 15 sec, 40 cycles. Melting curves were analyzed to verify single species PCR product. Fluorescent data were acquired at the 60°C. A template-free negative control was set for all experiments. GAPDH was used as an internal control for sample normalization. Relative expressions of PGIS and IP mRNA were calculated as: ΔΔCt = ΔCt (Al-treated group)-ΔCt (control group).

### Western blotting of IP protein expression

Hippocampus (50 mg) was added with 0.5 ml of tissue lysate solution for protein extraction and centrifugation at 12,000 × *g* for 10 min at 4°C, and the supernatant was used for Western blotting. The hippocampal protein content was detected with the bicinchoninic acid (BCA) method according to the directions of the protein detection kit.

Then,10% separating gel and 5% stacking gel were prepared for separation of proteins using sodium dodecyl sulfate polyacrylamide gel electrophoresis (SDS-PAGE). Protein was transferred to a polyvinylidene difluoride (PVDF) membrane. The membrane was blocked for 1.0 h using PBS containing 5% fat-free milk (weight/volume). The blot was incubated overnight at 4°C with primary antibodies at 1:300 for IP and at 1:1000 dilutions for β-actin, and then was incubated for 2 h at 37°C with a secondary antibody at 1:2000 for β-actin and IP. Immunoreactive bands of IP and β-actin were visualized with a BeyoECL plus chemiluminescence detection kit, and the optical density of bands was detected by a BioRad gel imaging and analysis system. The protein level of IP was calculated as ratios of the corresponding β-actin protein level.

### Statistical analysis

Data were presented as means ± standard deviation (SD) and analyzed on SPSS 17.0. Between-group differences in behavior test were evaluated by two-way analysis of variance (ANOVA), and other between-group differences were evaluated by one-way ANOVA; the Al-treated group and the control group were compared by Dunnett’s t-test. Results were considered significant with *P* < 0.05.

## Results

### Spatial learning and memory function of rats

Throughout the 20 weeks of experiments, a total of nine rats died, including two rats in control group, three rats in Al-overload group, two rats in BPS-6 group and two rats in BPS-12 group. However, autopsy results of dead rats showed no obvious abnormality in peripheral tissues or organs.

Compared to the control group, at the stage of spatial learning function test from d 3 to d 4, the time for the Al-overload group to learn to navigate the maze significantly increased (p < 0.05); on d 5 at the stage of spatial memory function test, the exploring time was significantly longer, p < 0.01. Administration of BPS significantly reduced the exploring time of aluminum-overload rats (Table 1).

**Table 1:** **Effects of BPS on change of spatial learning and memory functions in aluminum overload rats**

	**N**	**Exploring time(s)**				
		**d 1**	**d 2**	**d 3**	**d 4**	**d 5**
Control group	13	60.5 ± 17.4	40.1 ± 10.8	25.0 ± 11.3	19.7 ± 11.9	11.1 ± 6.5
Al-overload group	12	62.5 ± 15.7	49.1 ± 12.7	41.7 ± 19.1^#^	33.0 ± 11.8^#^	30.4 ± 17.7^##^
BPS-6 group	13	61.8 ± 15.5	39.6 ± 18.8	35.0 ± 16.6	27.4 ± 4.5	17.6 ± 8.5^*^
BPS-12 group	13	59.8 ± 18.3	36.3 ± 13.9	25.4 ± 6.9	21.1 ± 8.2^*^	14.3 ± 6.4^**^
BPS-24 group	15	62.6 ± 15.8	39.3 ± 13.2	31.4 ± 11.9	23.8 ± 9.9^*^	15.4 ± 8.7^*^

Data are expressed as mean ± SD of individual experiments. ^#^P < 0.05 and ^##^P < 0.01 compared with control group; ^*^P < 0.05 and ^**^P < 0.01 compared with Al-overload group.

### Morphological change of rat hippocampus

**Figure 1:**
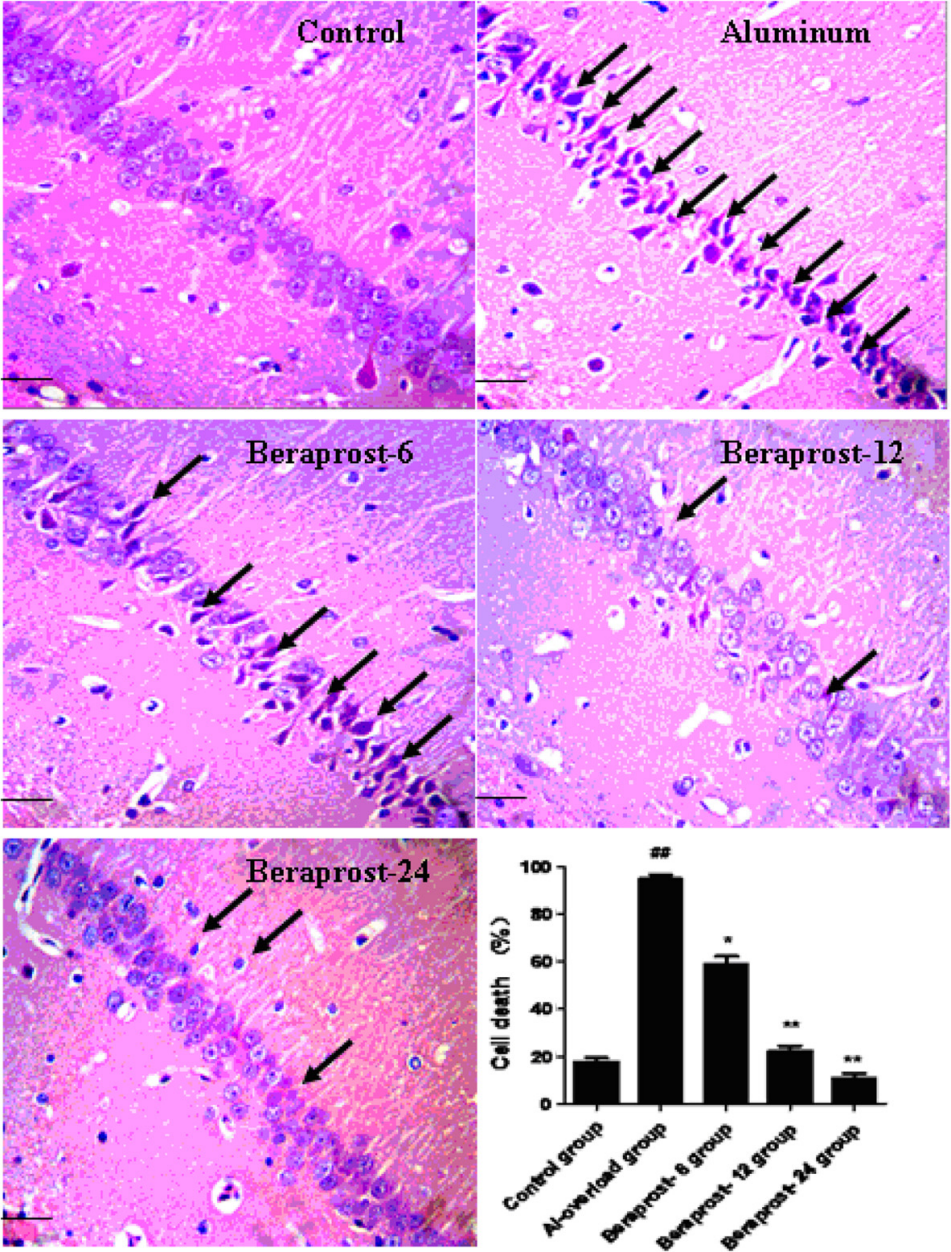
**Morphological change of rat hippocampal CA1 subfield neuron induced by aluminum overload.** HE staining, 400×. Scale bars = 50 μm. Arrow indicates karyopyknosis of cells. Dead nerve cell is characterized by eosinophilic change including nerve cell became a deep and red cell with nuclear pyknosis and nucleoli disappear under light microscope. ^##^P < 0.01 compared with control group; ^*^P < 0.05 and ^**^P < 0.01 compared with Al-overload group (mean ± SD, n = 4).

Hippocampal nerve cells in the control group were closely arranged and well structured, and the morphological structure kept intact and clear. The hippocampal CA1 subfield neurons in Al-overload group showed significant karyopyknosis and loss. The administration of BPS significantly prevented the histopathological changes of hippocampal neurons caused by aluminum overload in rats (Figure 1).

### SOD activity and MDA content in rat hippocampus

MDA content increased and SOD activity decreased both significantly in Al-overload rats compared to the control group. BPS administration significantly blunted the increase of MDA content and the decrease of SOD activity induced by aluminum (Table 2).

**Table 2:** **Effects of BPS on change of MDA content and SOD activity induced by aluminum overload in rat hippocampus (n = 4)**

	**MDA(nmol/mg protein)**	**SOD(U/mg protein)**
Control group	0.92 ± 0.09	13.54 ± 2.02
Al-overload group	4.45 ± 1.02^##^	7.98 ± 1.29^##^
BPS-6 group	2.92 ± 0.23^*^	9.01 ± 2.55
BPS-12 group	2.75 ± 0.49^*^	11.31 ± 1.20^*^
BPS-24 group	2.36 ± 0.70^*^	11.09 ± 0.90^**^

Data are expressed as mean ± SD of four individual experiments. ^##^P < 0.01 compared with control group; ^*^P < 0.05 and ^**^P < 0.01 compared with Al-overload group (n = 4).

### Level of 6-k-PGF_1α_ in rat hippocampus

The 6-k-PGF1α level in the hippocampus significantly increased in the Al-overload rats. Treatment of BPS significantly blunted the effect of aluminum on 6-k-PGF1α level in hippocampus (Table 3).

**Table 3:** **Effects of BPS on change of 6-k-PGF1α content induced by aluminum overload in rat hippocampus (n = 4)**

	**6-k-PGF1α(ng/g tissue)**
Control group	6.70 ± 1.28
Al-overload group	12.16 ± 1.35^##^
BPS-6 group	6.99 ± 0.50^**^
BPS-12 group	7.82 ± 0.31^*^
BPS-24 group	7.38 ± 1.08^*^

Data are expressed as mean ± SD of four individual experiments. ^##^P < 0.01 compared with control group; ^*^P < 0.05 and ^**^P < 0.01 compared with Al-overload group (n = 4).

### Expression of PGIS mRNA in rat hippocampus

The qRT-PCR analysis showed that aluminum overload significantly increased PGIS mRNA expression in rat hippocampus. The BPS significantly attenuated the aluminum-induced increase of PGIS mRNA expression in rat hippocampus (Table 4).

**Table 4:** **Effects of BPS on change of PGIS mRNA expression induced by aluminum overload in rat hippocampus (n = 4)**

	**The relative mRNA expression (PGIS/GADPH)**
Control group	1.00 ± 0.10
Al-overload group	5.05 ± 0.21^##^
BPS-6 group	3.27 ± 0.54^*^
BPS-12 group	2.42 ± 0.25^**^
BPS-24 group	1.97 ± 0.11^**^

Data are expressed as mean ± SD of four individual experiments. ^##^P < 0.01 compared with control group; ^*^P < 0.05 and ^**^P < 0.01 compared with Al-overload group (n = 4).

### Expressions of IP mRNA and protein in rat hippocampus

Compared to the control group, hippocampal IP mRNA level in aluminum-treated group significantly increased. Administration of BPS significantly down regulated the IP mRNA expression in Al-overload rat hippocampus (Table 5).

**Table 5:** **Effects of BPS on change of IP mRNA expression induced by aluminum overload in rat hippocampus (n = 4)**

	**The relative mRNA expression (IP/GADPH)**
Control group	1.00 ± 0.10
Al-overload group	2.21 ± 0.22^##^
BPS-6 group	1.90 ± 0.02^*^
BPS-12 group	1.58 ± 0.05^**^
BPS-24 group	1.36 ± 0.11^**^

Data are expressed as mean ± SD of four individual experiments. ^##^P < 0.01 compared with control group; ^*^P < 0.05 and ^**^P < 0.01 compared with Al-overload group (n = 4).

**Figure 2:**
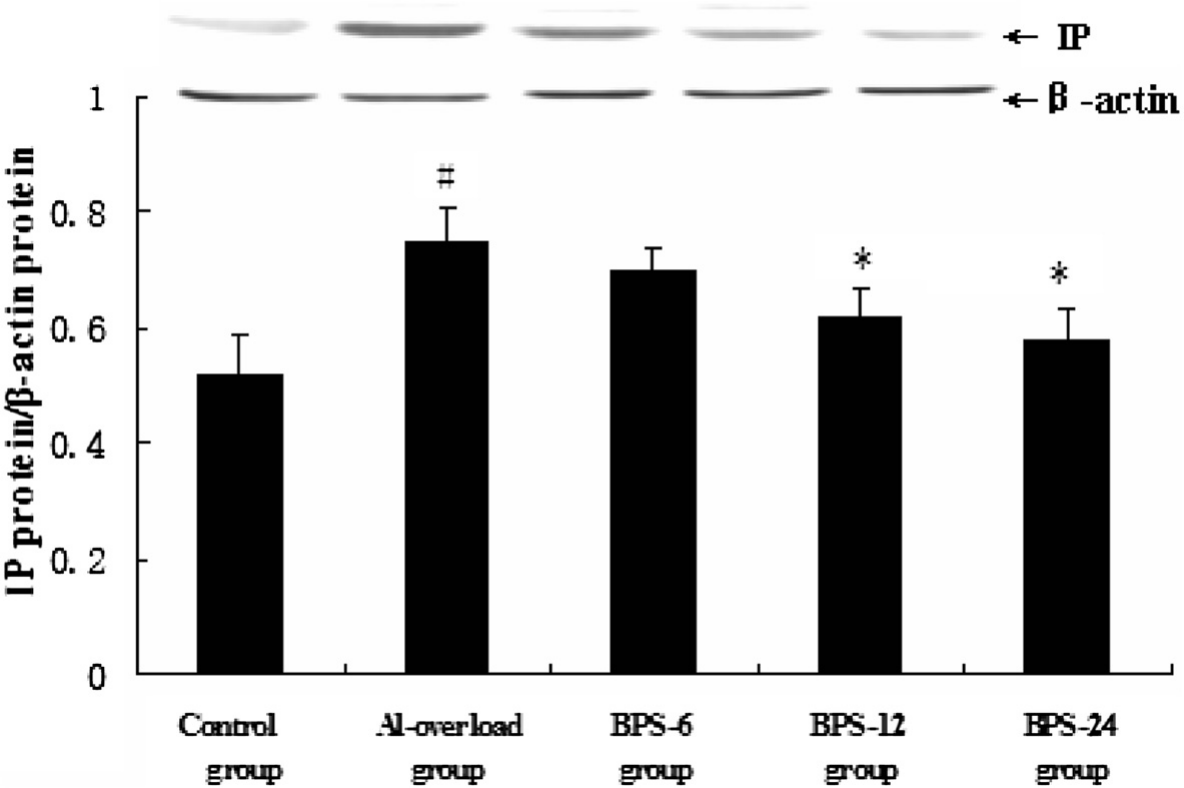
**Effects of BPS on change of IP protein expression induced by aluminum overload in rat hippocampus (n = 4).** The relative protein level of IP was normalized to endogenous β-actin protein for each sample. Data are expressed as mean ± SD of four individual experiments. ^#^P < 0.05 compared with control group; *P < 0.05 compared with Al-overload group.

The Western blot analysis showed that IP protein level in the hippocampus was significantly higher in aluminum-treated rats compared with the control group. Treatment with BPS significantly reduced the aluminum-induced increase of the IP protein expression in rats (Figure 2).

## Discussion

Episodic memory is a type of long-term memory based on personal daily events and factual knowledge, which is the most clinically relevant memory system. Episodic memory depends on the limbic system, hippocampus and frontal lobes. Any morphological alteration of hippocampal neurons will result in cognitive impairment, which is the most common hallmark for brain injury and neurodegeneration [18]. Therefore, in this study, we observed the change of histopathology in chronic aluminium gluconate-overload rat hippocampi. The present study results showed that chronic administration of aluminium gluconate resulted in the impairment of spatial learning function and spatial memory function and hippocampal neuron injury in rats. This is similar with our previous reported results [8,15].

Prostacyclin synthase (PGIS) converts the PGH2 derived from arachidonic acid *via* COX to PGI2 and produces important physiological function by acting mainly on IP. Besides endothelial cells, PGIS is also predominantly expressed in cortex and hippocampus neurons companied with IP expression. Cerebral ischemia reperfusion injury will result in a delayed and transient induction of PGIS in cortex and hippocampus neurons [19]. COX-1/PGIS adenoviral gene transfer to substantia nigra can prevent 6-OHDA-induced dopamine depletion and behavioral deficits in rats [20]. Combined gene transfer of COX-1 and PGIS also can increase PGI2 level and reduce cerebral infarct volume [12]. IP-/-mice compared with wide type mice show significantly larger infarct volume and greater behavioral damage, and administration of beraprost can significantly improve the neurological deficits and decrease the infarct volume in wild type mice [21]. These previous studies indicate that activation of PGIS/IP signaling is neuroprotective for acute traumatic brain injury and cerebral ischemic reperfusion insult. However, potential value of such activation for chronic brain injury is still not clear. We are the first to find that compared with the control group, PGIS mRNA expression, PGI2 level and the IP mRNA and protein expressions significantly increased in hippocampus of chronic aluminum-overload rats. Our results suggest that there is activation of PGIS/IP signaling pathway in chronic brain injury rat hippocampus subjected to aluminum overload. However, our studies showed that although the expression levels of IP in the hippocampus were significantly induced, the rats still displayed obvious spatial learning and memory impairment and obvious hippocampal neurons injury. These observations may be due to the increase of IP levels being insufficient to antagonize the injury caused by aluminum overload. Therefore, exogenous IP agonists should be supplied.

Our experiments also showed that the administration of BPS, which is an agonist of IP, could significantly improve spatial learning and memory function impairment and hippocampal neuron injury in chronic aluminum overload rats. Meanwhile, the administration of BPS resulted in a decrease of PGI2 level and a downregulation of PGIS and IP expressions. Our results suggest that BPS has a protective effect on cerebral injury induced by chronic aluminum overload in rats. Oxidative metabolism can supply energy to neurons. At the same time, this process may produce a lot of reactive compounds such as oxyradicals and hydrogen peroxide, which may cause peroxidation of biomembrane lipids, DNA damage, and neuronal death. The present study results showed that aluminum gluconate overload resulted in a significant decrease of SOD activity and a significant increase of MDA content. Our results further confirmed that oxidative stress contributed to aluminum gluconate induced chronic brain injury. Our previous studies showed that chronic aluminium gluconate exposure caused significant increase of Al, Fe, Mn, Cu and Zn level in rat hippocampus [9]. The brain accumulation of these metals (Al, Fe, Mn, Cu and Zn) could cause a significant oxidative stress. The latter cause the tissue damage and formation of AA by degradation of cell membrane phospholipids. BPS administration also significantly blunted the decrease of SOD activity and the increase of MDA content induced by aluminum overload in rats.

About the neuroprotective mechanisms of BPS, considering it can relieve oxidative stress in aluminum-overload rat, the effect of BPS on lipid peroxide formation may involve in the neuroprotective mechanisms. Previous research reported that BPS could relieve the ischemic and recirculation-induced cerebral injury through inhibition of lipid peroxide formation [22]. Besides this, BPS can relieve oxidative stress injury and inhibit the neuroinflammation in CNS by stimulating the IP receptor and in turn results in decrease of cell membrane phospholipids degradation and PGH2 formation. The decrease of PGH2 level might cause the decrease of PGIS expression and PGI2 content. On the other hand, a long-term use of BPS also caused downregulation of IP mRNA and protein expressions by a negative feedback regulation mechanism of receptor.

Together with these results, our experimental results suggest that the PGIS/IP signaling pathway in chronic brain injury rat hippocampus is activated and that beraprost sodium has a neuroprotective effect on chronic brain injury through remodeling the balance of PGIS/IP signaling pathway and inhibition of oxidative stress in aluminum-overload rats. The PGIS/IP signaling pathway is promising to potential therapeutic strategy for chronic brain injury patients. However,the effect of BSP on IP downstream signaling pathway is necessary to further study in detail.

## Abbreviations

Al: Aluminum
AD: Alzheimer’s disease
IL: Interleukin
TNF: Tumor necrosis factor
AA: Arachidonic acid
PLA2: Phosphalipase A2
PG: Prostaglandin
COX: Cyclooxygenase
TXA2: Thromboxane A2
NSAIDs: Non-steroidal antiinflammatory drugs
IP: Prostacyclin receptor
PGI2: Prostacyclin
PGIS: Prostacyclin synthase
BPS: Beraprost sodium
CNS: Central nervous system
BCA: Bicinchoninic acid
H&E: Hematoxylin and eosin staining
MDA: Malondialdehyde
SOD: Superoxide dismutase
ELISA: Enzyme-linked immunosorbent assay
CMC-Na: Sodium carboxy methyl cellulose
qRT-PCR: quantitative real-time polymerase chain reaction
PVDF: Polyvinylidene difluoride
SD: Standard deviation
ANOVA: Analysis of variance
GAPDH: Glyceraldehyde phosphate dehydrogenase
